# Responsible leadership and project citizenship behavior: A cross-level investigation

**DOI:** 10.3389/fpsyg.2022.960290

**Published:** 2022-09-02

**Authors:** Yuxin Yang, Jieying Huang, Pingping Wu, Xujiang Zheng, Han Lin, Shule Ji

**Affiliations:** ^1^Jiangsu Key Laboratory of Public Project Audit, School of Engineering Audit, Nanjing Audit University, Nanjing, China; ^2^McCormick School of Engineering, Northwestern University, Evanston, IL, United States; ^3^School of Law, Zhejiang Sci-Tech University, Hangzhou, China; ^4^Institute of Law, Chinese Academy of Social Sciences, Beijing, China

**Keywords:** responsible leadership, project citizenship behavior, moral identity, collective moral sensitivity, construction project

## Abstract

Project citizenship behavior (PCB) has an important positive impact on project success. Researching how to promote PCB is an important issue in project management. Based on social learning theory and social cognitive theory, this paper adopted the method of questionnaire survey and hierarchical linear model (HLM) to analyze the collected data derived from the sample of Chinese construction enterprises and verified this hypothesis. The results show that responsible leadership has a significant positive effect on PCB, moral identity mediates this relationship, and collective moral sensitivity positively moderates this mediating effect. The findings of the study systematically and deeply reveal the intrinsic mechanism of the cross-level influence of responsible leadership on PCB, and provide new enlightenment for the practice of project management.

## Introduction

Project teams have become more and more important in the construction projects (Wang et al., 2020; Ni et al., 2022). An excellent project team cannot only maintain a harmonious project atmosphere and ensure the safety and quality of the project construction, but even complete the project ahead of schedule. According to previous studies, leadership is an important factor influencing team performance and competitive advantage through the influence and guidance of team members (Lee et al., 2010; Rao and Abdul, 2015; Zaim et al., 2015). Although there is a large body of researches on leadership, most of them have focused on ethical leadership, transformational leadership, authoritative leadership, etc., and few of these researches have focused on the specific environment and conditions of the organization. Given the critical role that leadership plays in project teams, more researches on leadership are needed.

Responsible leadership is defined as the art and ability to establish, cultivate, and maintain trusting relationships with stakeholders both inside and outside the organization, as well as the responsibility behavior to work together to achieve a meaningful and shared business vision (Maak and Pless, 2006; Maak, 2007). Scholars believe that responsible leadership has a significant impact on employees’ attitude and behavior (Brown, 2006; Voegtlin et al., 2012). Responsible leadership will be paid attention to by employees, directly or indirectly affect employees’ attitude and cognition, and then affect organizational citizenship behavior (OCB) of employees (Lord and Brown, 2001). Although empirical researches have proved that responsible leadership is positively correlated with OCB of employees, most of them focus on OCB for the environment or in the hospitality industry (Han et al., 2019). As far as we know, no research has investigated the relationship between responsible leadership and citizenship behavior in the context of project environment. Different from general organizations, construction projects have the characteristics of temporariness, team, task, and context-embeddedness (Braun et al., 2012; Xia et al., 2018), which requires us to pay more attention to the perspective of temporary organizations when carrying out relevant research. Our study attempts to fill this research gap.

The theory of project citizenship behavior (PCB) is developed from the concept of OCB (Wang et al., 2021). PCB is considered to be the positive behavior carried out by construction participators in the project, which is not within the scope of the contract, but contributes to the realization of the project objectives as a whole (Yang et al., 2018). The research shows that the success of construction projects depends not only on the external conditions of project construction, but also on the citizenship behavior of the project participants. In the process of construction project construction, the highly subjective initiative and creativity demonstrated by PCB can promote the project team to make behaviors beyond the scope of normal contract, and work more efficiently in the complex and uncertain construction project site. In view of the important impact of PCB on the implementation of project, it is necessary to research how and when the responsible leadership influences PCB.

Social learning theory suggests that individuals can guide their own behavior by observing, learning, and imitating the behavior of others (Bandura and Walters, 1977; Mayer et al., 2009). In the projects, team members gradually accept the leadership values by observing and imitating. They begin to show the attention to morality and responsibility and focus on the realization of objectives and the overall interests of the project. This benign behavior is in conformity with the connotation of PCB. According to social cognitive theory (Bandura, 1991), the behavior of individuals is not only determined by their own characteristics, but also influenced by their environment. As an important factor in the subordinate’s environment, responsible leadership can greatly influence the behavior of project members. Responsible leadership can influence PCB based on these two concepts.

Previous studies on responsible leadership and OCB have selected organizational identification and leader identification as mediating variables, while ignoring moral identity. Moral identity is defined as a group of cognitive schemata centered on moral qualities, including moral values, goals, and behavior scripts (Narvaez and Lapsley, 2009) and it is an important basis for individuals to understand and engage moral behavior (Hardy and Carlo, 2011). As described in the social learning theory (Mayer et al., 2009), individuals imitate leaders by observing and learning. In the projects, subordinates take responsible leaders as models and gradually internalize their sense of responsibility and morality. This provides the basis for the formation of moral identity. At the same time, the moral driving force derived from moral identity will contribute to the generation of PCB. Based on the above discussion, we propose that moral identity plays a mediating role between responsible leadership and PCB.

Moral sensitivity is defined as the ability of an individual to recognize the impact of one’s behavior on others in a certain situation and the moral problems involved (Bebeau et al., 1985; Sadler, 2004). According to social cognitive theory (Bandura, 1991), the cognitive level affects behavioral response. Teams with high collective moral sensitivity are more likely to pay attention to moral characteristics (Lovett and Jordan, 2010), which is reflected in the attention and identity of the social responsibility and morality of responsible leaders. This will push project members to give feedback to the project and facilitate PCB more easily. Therefore, we argue that collective moral sensitivity moderates the effect of moral identity on PCB.

On this basis, this paper aims to better understand the mechanisms by which and conditions under which, responsible leadership can effectively promote PCB. The theoretical model of this paper proposes that responsible leadership can effectively promote PCB, moral identity plays a mediating role in this relationship. This mechanism is also moderated by the collective moral sensitivity. The data analysis and verification of Chinese construction firms prove these hypotheses. This paper has enriched the research perspective of citizenship behavior in the field of construction projects, expanded the theoretical research of leadership on PCB, revealed the internal mechanism of responsible leadership on PCB, and provided new enlightenment and important guidance for the practice of construction project management.

## Theoretical background and research hypotheses

### Responsible leadership

In recent years, under the circumstance of the complex and changeable economic environment and increasingly fierce market competition, in order to promote the development of organizations, leaders need to pay attention not only to economic interests, but also to the relationship with stakeholders and the corporate social responsibility especially in construction projects (Zeng et al., 2015; Lin et al., 2017, 2018; Ma et al., 2017). Responsible leadership represents a powerful response to the demands of market competition and economic environment. The research on responsible leadership originates from the combination of social responsibility and leadership behavior (Maak and Pless, 2006). It is the leadership behavior that leaders seek to establish, cultivate, and maintain a long-term trust relationship with stakeholders inside and outside the organization in order to achieve a harmonious and win-win situation among enterprises, society, and the environment (Maak and Pless, 2006). Responsible leadership occupies a central position in the stakeholder relationship network and also plays a variety of roles. It achieves consensus on the requirements of stakeholders through democratic consultation and conversation (Voegtlin et al., 2012). It involves the establishment and maintenance of good relations with all stakeholders. The main difference between responsible leadership and other traditional forms of leadership (e.g., transformational leadership, moral leadership, service leadership, and authoritative leadership) is that it attaches importance to the interest appeal of stakeholders, social responsibility, and sustainable development. Traditional forms of leadership, such as moral leadership, overemphasize its impact and fail to fully recognize its circumstance and environment and the interactions of other stakeholders. Moreover, these forms of leadership ignore the dimension of responsibility. Responsible leadership makes up for these defects, transcends the binary leader-follower relationship in traditional leadership, and emphasizes the relationship between leaders and stakeholders inside or outside organizations. Specific to the project area, responsible leadership pays attention to safeguarding the interests of all stakeholders in decision-making and long-term development of the project. It focuses on the leader’s own responsibility and the role in the project.

Regarding the impact of responsible leadership, the literature generally supports two types of impact, namely, the impact on the organization and the impact on its employees. According to the research, at the organizational level, responsible leadership can make enterprises undertake more social responsibilities, thus promoting the sustainable development of enterprises (Székely and Knirsch, 2005). At the employee level, responsible leadership can enhance the pride and job satisfaction of employees, and the behavior of responsible leaders will serve as a model for employees. Increase employees’ loyalty to the organization (Doh et al., 2011). In general, responsible leadership affects employees’ attitudes and perceptions, such as job satisfaction, job meaning, and OCB (Doh et al., 2011; Voegtlin et al., 2012).

### Project citizenship behavior

The theory of PCB is derived from the extension of the concept of OCB to project management. OCB is defined as “the behavior that employees take the initiative to promote the development of the organization based on their own understanding, and this behavior is not mandated by the organization, nor is there any reward or remuneration” (Bateman and Organ, 1983). Research also pointed out that citizenship behavior has potential value in less restrictive organizations (Bateman and Organ, 1983). These organizations are increasingly project-oriented (Huemann et al., 2007; Xia et al., 2018). With the deepening of the research on citizenship behavior, Braun et al. (2012) put forward the concept of PCB according to the characteristics of the project when exploring the citizenship behavior of the project as a temporary system, they proposed that PCB is the cooperative behavior of project staff beyond the contractual requirements in a specific project. PCB is a multidimensional concept, including five interrelated behaviors, namely, helping behavior, project loyalty, project compliance, individual initiative, and relationship maintenance (Braun et al., 2013). Helping behavior is a kind of spontaneous behavior that helps project members solve problems, project loyalty means that project members are loyal to the project, project compliance refers to project members’ compliance with relevant regulations and quality standards, personal initiative refers to task-related behaviors that exceed minimum requirements and relationship maintenance refers to the behavior of maintaining the relationship between project members even after the termination of the existing project (Braun et al., 2013).

Project citizenship behavior differs from OCB mainly in time, task, team, and context (Braun et al., 2012). Time is the most critical feature that distinguishes a project from a permanent organization (Braun et al., 2012). A project has a specific construction cycle, during which the project must rely on the temporary project team (Bakker, 2010), which is disbanded at the end of the project, while the general organization lasts for a long time. At the same time, a project is connected with a wider social context, so there are essential differences between PCB and OCB. In addition, the characteristics offered by the project team structure and the non-repetitive tasks which are more likely in the projects may stimulate the evolution of PCB compared to ordinary organizations (Braun et al., 2012). Although there is no clear stipulation in the construction contract, the research shows that the success of the project depends not only on the external conditions of the project construction, but also on the citizenship behavior of the participants (He et al., 2015; Yang et al., 2018).

### Responsible leadership and project citizenship behavior

Social learning theory emphasizes the demonstration effect of role models. Followers can understand how to behave both by observing the behavior of others and through their own experiences (Bandura and Walters, 1977). Leaders are the focus of employees’ attention, and employees learn by observing leaders, so as to imitate their behavior (Mayer et al., 2009). Research shows that responsible leaders can improve the moral idea and the sense of responsibility of their subordinates (Haque et al., 2019). Leaders influence their subordinates more effectively when subordinates regard the leader as a model of the group and representative of the whole group (Gerpott et al., 2019). In the construction projects, by setting the example of being responsible, honest, moral, and paying attention to stakeholders, responsible leaders can enhance employees’ trust and recognition of leaders and stimulate imitation motivation. Thus, subordinates are able to change and innovate by internalizing what they perceive and engage the behavior that promotes the development of the project.

According to social cognitive theory, the behavior of individuals is not only determined by their own characteristics, but also influenced by their environment, i.e., by the social psychology of self-perception when individuals are in organizations (Wood and Bandura, 1989). As an important part of the organizational environment (Kuenzi et al., 2020), leaders play a key role in the operation of the project and they are very critical in the process of supporting and motivating subordinates to promote “extra-role” behavior. Therefore, as an important factor in the subordinate’s environment, responsible leadership can greatly influence the behavior of project members. Specifically, responsible leaders encourage subordinates to participate in the decision-making process and promote a sense of ownership, thereby stimulating PCB.

In short, under the influence of responsible leaders in construction projects, project members are more likely to carry out positive behavior that are not within the scope of the contract but contribute to the realization of the project objectives as a whole, namely, PCB. Therefore, we propose:

Hypothesis 1: Responsible leadership has a positive effect on project citizenship behavior.

### Mediating role of moral identity

Beyond the direct effects of responsible leadership on PCB, we suggest that the above relationship will be mediated by moral identity at least in part. Moral identity is an individual self-definition based on moral traits such as caring, equality, and helping others, and it is a self-regulating mechanism that guides moral judgment and moral behavior (Mayer et al., 2012). Research suggests that moral identity may be an important source of moral drive, and moral identity may be the best predictor of moral commitment and moral behavior (Hertz and Krettenauer, 2016). Moral identity helps restrain individual unethical behavior and promote individual moral behavior (Aquino et al., 2007).

According to social learning theory, individuals can observe and learn to imitate leaders. When responsible leaders pay attention to the environment and conditions of the project, fully consider the relationship between stakeholders inside and outside the project, and pursue long-term interests and sustainable development, they will set examples to their subordinates. Subordinates will regard leaders as the model in the team and by learning and imitating they will gradually internalize the responsibility and morality of responsible leaders and make their own moral values tend to responsible leaders, thus providing a certain basis for the formation of moral identity. At the same time, moral identity is the key psychological mechanism that transforms moral cognition into moral behavior (Aquino and Reed, 2002; Hertz and Krettenauer, 2016). People with high level of moral identity are more likely to show their moral character and gain recognition from others (Jennings et al., 2015). After identifying and internalizing the morality of responsible leadership, project members tend to be motivated to engage in behaviors that promote the development of the project. Therefore, the moral driving force derived from moral identity will contribute to the generation of PCB.

In short, we believe that moral identity is one of the important factors to ensure that responsible leadership promotes PCB. When employees have high moral identity, they can easily distinguish the behaviors advocated by responsible leadership, learn and emulate them, and promote PCB. Therefore, we propose the following hypothesis:

Hypothesis 2: Moral identity plays a mediating role in the positive relationship between responsible leadership and project citizenship behavior.

### Moderating role of collective moral sensitivity

Moral sensitivity refers to the ability of an individual to recognize the impact of one’s behavior on others in a certain situation and to recognize the moral problems involved (Bebeau et al., 1985; Sadler, 2004). Moral behavior is the combined action of moral sensitivity, moral judgment, moral motivation, and moral character.

Social cognition theory emphasizes that the individual’s cognitive level affects his behavioral response (Bandura, 1991). High moral sensitivity helps individuals enhance the ability to identify and deal with moral and ethical issues, and they tend to be active bystanders. Low moral sensitivity leads to moral disregard and moral indifference, and individuals tend to be passive bystanders (Fowler et al., 2009; Pedersen, 2009; Nora et al., 2017). Generally speaking, moral sensitivity is studied from the perspective of individual differences, but previous researches have proved that people can obtain characteristics from the environment through the perception of environmental factors (Park and Deshon, 2010; Dragoni and Kuenzi, 2012). When team members are exposed to the same environmental factors, they tend to reach a consensus on the identification and detection of moral issues, which will also be reflected in the collective moral sensitivity. Based on the research of moral sensitivity and moral behavior, we believe that the moral identity affect PCB may vary with the level of collective moral sensitivity, high collective moral sensitivity means that employees have a greater sense of moral and social responsibility and their motivation and behavior tend to be more susceptible to the influence of moral identity. Teams with high collective moral sensitivity are more likely to pay attention to moral characteristics (Lovett and Jordan, 2010), which is reflected in the attention and identity of the social responsibility and morality of responsible leaders. This recognition will push project members to give feedback to the project and facilitate PCB more easily. In contrast, teams with low collective moral sensitivity tend to be indifferent to moral issues in construction projects, and even ignore or disagree with the morality and responsibility of the responsible leadership, so they less likely to engage PCB. Based on the above views, we propose the following hypothesis:

Hypothesis 3: Collective moral sensitivity positively moderates the mediating effect of moral identity on the relationship between responsible leadership and project citizenship behavior.

**FIGURE 1:**
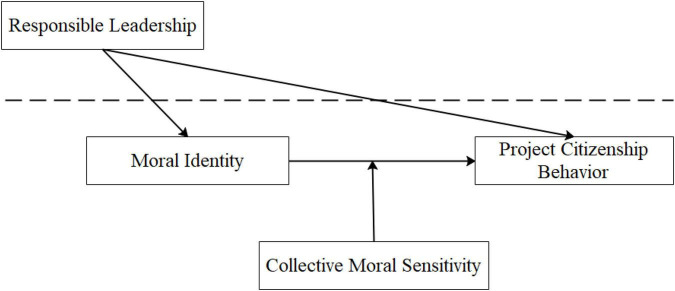
The conceptual model.

Our conceptual model is presented in Figure 1.

## Methodology

### Sample and data collection

To test the hypotheses presented in the previous section, for the field of construction industry, this study used questionnaires that were executed according to the standard steps of the research design in order to ensure validity and avoid cultural bias (Lin et al., 2018; Yu et al., 2019). Since the original items were all in English, two scholars with rich research experience in related fields were invited to translate the scales into Chinese then back into English. Moreover, this study adopted *ex ante* procedural remedies to reduce bias that common methods can introduce (Podsakoff et al., 2003). The questionnaire consisted of two parts. The part 1 contained variables related to the company and the specific project and was completed by project managers, and the part 2 contained variables related to the individuals and was completed by project members in the specific project. It was clearly stated that there were no right or wrong choices and informant anonymity was guaranteed to protect the privacy (Wei et al., 2018).

This study was conducted with four experienced academics and four professional managers to verify the content, phraseology, and clarity of the draft, and to revise or delete all question items to obtain the final version. First, we contacted the China Association of Construction Enterprises (CACE) and obtained a list of its members. Then we selected companies located in China’s Yangtze River Delta as the sample for the study. Thereafter, we randomly contacted these companies and distributed the questionnaire to the participants on site. Finally, a total of 48 companies, 92 project groups and 555 respondents were included in the final sample. Of these 92 project groups, 78.26% of project managers were male; 6.52% of managers had a middle school degree, 15.22% had a college degree, 58.68% had a bachelor degree, and 8.70% had a master degree; 4.35% of project managers were under 29 years old, 15.22% were between 30 and 34 years old, 46.74% were above 35 and below 39 years old, 33.70% were over 40 years old; the mean of project tenure is 6.45 years (SD = 5.04) and the mean of company tenure is 9.22 years (SD = 4.51).

### Measurements

Unless otherwise specified, all perceptual items were assessed on five-point Likert-type scales ranging from 1 (strongly disagree) to 5 (strongly agree). To measure the variable for multi-item constructs, average scores were calculated.

#### Responsible leadership

In line with Voegtlin (2011), responsible leadership was measured from five items: My direct supervisor demonstrates awareness of the relevant stakeholder claims; My direct supervisor considers the consequences of decisions for the affected stakeholders; My direct supervisor involves the affected stakeholders in the decision making process; My direct supervisor weighs different stakeholders claims before making a decision and My direct supervisor tries to achieve a consensus among the affected stakeholders. Cronbach’s alpha coefficient for responsible leadership was 0.86.

#### Project citizenship behavior

To measure the PCB, a twenty-item scale adopted from Braun et al. (2013) was employed. The sample items are “I help project staff when they have heavy workloads.”, “I offer the project team members a helping hand if they need it at some stage in the course of the project.”, and “I intervene and try to balance interests when disputes in the project team occur.” Cronbach’s alpha coefficient for PCB was 0.85.

#### Moral identity

The authors followed the method developed by Aquino and Reed (2002) to measure the moral identity. It was measured from ten items. The sample items are “I often wear clothes that identity me as having these characteristics.” and “The types of things I do in my spare time (e.g., hobbies) clearly identity me as having these characteristics.” Cronbach’s alpha coefficient for moral identity was 0.90.

#### Collective moral sensitivity

This study adopted seven measurement items of collective moral sensitivity purposed by Arnaud (2010). Sample items include “People in my department sympathize with someone who is having difficulties in their jobs.” and “For the most part, when people around here see that someone is treated unfairly, they feel pity for that person.” Cronbach’s alpha coefficient for collective moral identity was 0.88.

#### Control variables

Based on previous literature (Sang et al., 2021), considering the potential influence of project managers’ personal situation (e.g., age, gender, education, company tenure, and project tenure) and to exclude alternative explanations, this research adopts five control variables. Project managers’ age was defined by integer values from 1 to 4 (1 = “below 29 years old,” 2 = “30–34 years old,” 3 = “35–39 years old,” 4 = “above 40 years old”), gender was represented by dummy variables (1 = male, 0 = female). Their education experience was expressed by numerical variables (1 = “a high school or technical secondary school degree,” 2 = “a college degree,” 3 = “a bachelor degree,” 4 = “a master degree,” 5 = “a doctor degree”). Company tenure and project tenure were described by project managers in years.

## Results

### Confirmatory factor analysis

As the baseline model with full items met the condition that sample size is 5 times more than measurement item, and the questionnaires adopted mature scales, Confirmatory factor analysis (CFAs) were applied to test construct validity in this study (Yu et al., 2019).

In the test of construct validity, the baseline model fitting of the four-factor model was compared with the other four alternative models, and the results are shown in Table 1. According to Table 1, the baseline model had the best fitting degree with the sample data, and the best construct validity among variables compared with the other four alternative models (χ^2^/*df* = 3.37, IFI = 0.94, TLI = 0.93, CFI = 0.94, SRMR = 0.05, RMSEA = 0.05, GFI = 0.94, NFI = 0.92). According to Fernandes-Jesus et al. (2016), acceptable model-data fit can be inferred from χ^2^/*df* < 5, CFI > 0.9, SMRM < 0.08, and RMSEA < 0.08.

**TABLE 1:** Comparison of measurement models.

Model	χ^2^/*df*	Δ χ^2^/Δ *df*	IFI	TLI	CFI	RMSEA	SRMR	GFI	NFI
Baseline model	3.37	–	0.94	0.93	0.94	0.05	0.05	0.94	0.92
Three factors-RL and MI were combined	4.79	360.63	0.91	0.90	0.91	0.08	0.19	0.92	0.90
Three factors-RL and CMS were combined	4.52	287.54	0.92	0.91	0.92	0.08	0.14	0.90	0.90
Two factors-RL, MI, and CMS were combined	5.46	537.76	0.90	0.89	0.90	0.09	0.24	0.89	0.90
One factor	3.45	6.44	0.93	0.93	0.94	0.07	0.06	0.88	0.90

RL, responsible leadership; MI, moral identity; CMS, collective moral sensitivity.

### Hierarchical linear modeling analysis

Descriptive statistics and correlation analysis were carried out according to the obtained data. The means, standard deviants, and correlations are shown in Table 2.

**TABLE 2:** Means, standard deviants, and correlations.

	Mean	SD	1	2	3	4	5	6	7	8
1. Age	4.11	0.81	1							
2. Gender	1.13	0.32	−0.12**	1						
3. Education	2.65	0.71	0.05	−0.01	1					
4. Project tenure	6.45	5.04	−0.01	0.33**	0.13**	1				
5. Company tenure	9.22	4.51	−0.06	0.64**	0.01	0.32**	1			
6. RL	3.99	0.77	−0.02	−0.05	0.11**	0.11**	−0.10*	1		
7. MI	4.11	1.14	−0.05	0.01	0.08**	−0.11**	0.01	0.27**	1	
8. CMS	3.89	0.85	−0.03	−0.10*	−0.08*	−0.16**	−0.04	0.14**	0.44**	1
9. PCB	3.92	1.01	−0.06	−0.02	0.11**	−0.03	0.02	0.24**	0.38**	0.44**

*p < 0.05, **p < 0.01, ***p < 0.001.

RL, responsible leadership; MI, moral identity; CMS, collective moral sensitivity; PCB, project citizenship behavior.

In this study, the data obtained conforms to the nested structure. Based on this data structure, a two-level hierarchical linear modeling (HLM) is selected to research relationships within and between hierarchical levels. It is a valid analytical technique (Hong and Kim, 2021). Adopting the HLM can provide more accurate estimates of higher-level implementation (e.g., organizational level) on lower-level outcomes (e.g., individual level) (Sang et al., 2021). The results are presented in Table 3.

**TABLE 3:** Results of hierarchical linear model (HLM) analysis.

	MI		PCB					
		
	Model 1	Model 2	Model 3	Model 4	Model 5	Model 6	Model 7	Model 8
Cont.	1.73*** (0.05)	1.72*** (0.05)	1.54*** (0.05)	1.53*** (0.05)	1.75*** (0.06)	1.75*** (0.06)	1.72*** (0.06)	1.79*** (0.06)
Age	−0.29** (0.14)	−0.29** (0.14)	−0.17 (0.16)	−0.17 (0.16)	0.01 (0.02)	0.01 (0.02)	−0.01 (0.02)	−0.01 (0.02)
Gender	−0.01 (0.02)	0.00 (0.02)	−0.05 (0.07)	−0.05 (0.07)	−0.04 (0.06)	−0.04 (0.06)	−0.04 (0.06)	−0.04 (0.06)
Education	0.16** (0.07)	0.15** (0.07)	0.14* (0.07)	0.14* (0.07)	0.05 (0.08)	0.05 (0.08)	0.05 (0.08)	0.04 (0.08)
Project tenure	−0.03** (0.02)	−0.03** (0.02)	−0.01 (0.01)	−0.01 (0.01)	0.00 (0.01)	0.00 (0.01)	0.00 (0.01)	0.00 (0.01)
Company tenure	0.02* (0.01)	0.02* (0.01)	0.01 (0.01)	0.01 (0.01)	0.00 (0.01)	0.00 (0.01)	0.00 (0.01)	0.00 (0.01)
RL		0.14** (0.07)		0.23* (0.12)	0.13 (0.10)			
MI					0.26*** (0.05)	0.27*** (0.07)	0.27*** (0.07)	0.25*** (0.06)
CMS							0.04 (0.04)	0.05 (0.04)
MI*CMS								0.08* (0.04)
*R* ^2^	0.54	0.52	0.60	0.59	0.63	0.63	0.67	0.67
Adjusted *R*^2^	0.29	0.27	0.36	0.35	0.40	0.40	0.45	0.45
Chi-square	576.76	554.84	522.07	509.69	749.14	749.14	542.00	541.00

*p < 0.05, **p < 0.01, ***p < 0.001.

RL, responsible leadership; MI, moral identity; CMS, collective moral sensitivity; PCB, project citizenship behavior.

As shown in Models 2 and 4, responsible leadership is positively correlated to both moral identity (Model 2, γ = 0.14, *p* < 0.01) and PCB (Model 4, γ = 0.23, *p* < 0.05). Therefore, H1 is verified. H2 predicts that moral identity mediates the relationship between responsible leadership and PCB. In order to test H2, both responsible leadership and moral identity were incorporated into the HLM, and the coefficient of responsible leadership changed from 0.23 (Model 4, *p* < 0.05) to 0.13 (Model 5, *p* > 0.05). The coefficient of moral identity remained significant (model 5, γ = 0.26, *p* < 0.001), indicating its possible mediating role. A Bootstrap test was employed for the mediation effect (Lin et al., 2020). The indirect effect of responsible leadership on PCB through moral identity was identified (*z* = 2.011, *p* < 0.05), thus H2 is verified.

**FIGURE 2:**
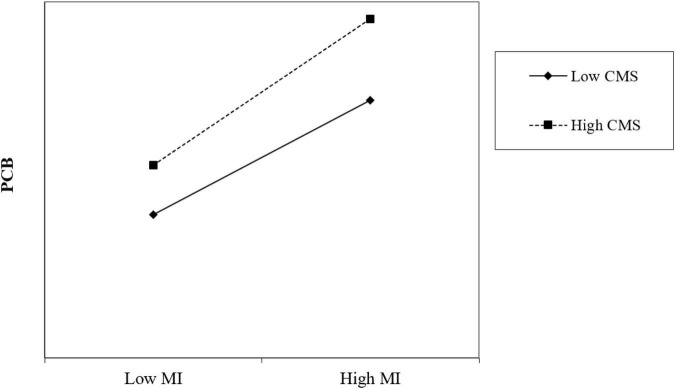
The moderating effect of collective moral sensitivity (CMS) on the relationship between moral identity (MI) and project citizenship behavior (PCB).

To test H3, the interactive term between moral identity and collective moral sensitivity is positively correlated with PCB (Model 8, γ = 0.08, *p* < 0.05), which is consistent with H3. In accordance with the procedure proposed by Stone and Hollenbeck (1989), the relationship between moral identity and PCB was plotted under the case of high and low collective moral sensitivity to further test this interactive effect on PCB. As shown in Figure 2, collective moral sensitivity strengthens the positive relationship between moral identity and PCB. Specifically, the positive association between moral identity and PCB will be strengthened when collective moral sensitivity is higher. We also conduct a bootstrap procedure with 5,000 random samples to calculate the indirect effects and confidence intervals of high level and low level collective moral sensitivity. The results show that the indirect effect of moral identity on responsible leadership and PCB is significant at 95% confidence level when collective moral sensitivity is high. In the case of low level collective moral sensitivity, the indirect effect of moral identity on responsible leadership and PCB is insignificant at 95% confidence level. Therefore, H3 is supported.

## Discussion and contribution

With the increasing awareness of safety in the construction industry, people pay more attention to the leaders who play a key role in construction projects since more and more evidence shows that different leadership styles lead to different behaviors of workers. Although this phenomenon has received a lot of attention, there is no relevant research to investigate the influence of responsible leadership on PCB. This study is the first to propose the cross-level impact of responsible leadership on PCB. The propose is verified and the influence mechanism is explore based on the questionnaire results of Chinese construction firms through theoretical analysis and empirical research. It is found that responsible leadership has a significant positive impact on PCB and moral identity plays a mediating role in this relationship. Meanwhile, collective moral sensitivity can moderate this mediating effect, namely, positive association between moral identity and PCB will be strengthened when collective moral sensitivity is stronger. These results provide new enlightenment not only for theoretical research but also for project management practice.

### Theoretical contributions

This study discusses cross-level impacts of responsible leadership on PCB and the main theoretical contributions are as follows.

First, this study enriches the research of the leadership theory. The concept of responsible leadership was born in the era of globalization. Its research duration is not long and there are still many research gaps to be solved by scholars. Following the call of Voegtlin et al. (2012) to carry out further research on responsible leadership, this paper applies social learning theory and social cognition theory, conducts empirical research on responsible leadership and makes contributions to the development of the responsible leadership theory. It not only helps scholars understand the theory of responsible leadership better, but also creates an important theoretical value for the broadening and perfecting of the research framework of leadership theory.

Second, the study broadens the boundaries of citizenship behavior research. While previous studies tend to analyze the influence of leadership style on citizenship behavior based on the perspective of general organizations, this paper explores the influence of responsible leadership on citizenship behavior from the perspective of construction project. Unlike general organizations, construction projects are temporary, complex, and uncertain and could be look upon as “one-off” projects (Zeng et al., 2015; Zhang et al., 2020; Sang et al., 2021). This study introduces a novel concept, PCB, which makes up for the limitations of previous research on citizenship behavior, and reveals the occurrence mechanism of citizenship behavior in a deeper level, fills the research gap of the influence of responsible leadership on citizenship behavior, and further improves the related theory.

Third, the study suggest that responsible leadership can promote PCB and that moral identity mediates this relationship, and the higher the collective moral sensitivity, the more pronounced the mediating role of moral identity in the relationship between responsible leadership and PCB. However, the facilitative effect of responsible leadership on PCB does not remain significant in all cases and cannot be studied without considering collective moral sensitivity, so it is particularly important to consider its contextual factors when conducting relevant studies. The results of this study supplement the theoretical basis of the positive impact of responsible leadership on PCB, provides a more detailed panorama of the relationship between responsible leadership and PCB, and clarifies the potential mechanism and constraints systematically and deeply.

Four, in the process of survey and questionnaire data analysis, due to the characteristics of nested structure between construction project organizations and individual workers, the study adopts a HLM different from the traditional linear model in previous studies to accommodate the characteristics of similar data at the same level and interactive data at different levels in the nested relationship, so as to describe the complex relationship of responsible leadership on PCB, analyze and process the corresponding data to ensure the accuracy, reliability, and validity of the research results and avoid the limitations of the traditional linear model. The data analysis ideas of the study can provide some new ideas for the subsequent data structure model analysis methods in the field of construction project management and the extended field for related issues.

### Managerial implications

In the practice of project management, the findings of this paper also provide management implications and important guidance for project managers. On the one hand, in the project, project managers are always in the key position of the organization, no matter how complex and varied the project organization is. Their perceptions of social responsibility and morality and their consideration of the interests and needs of various stakeholders will directly or indirectly influence PCB. When leaders demonstrate a strong sense of social responsibility and morality and concern for stakeholders in the organization, their positive guidance and role model will make subordinate workers identify with and learn from them, thus motivating subordinates to contribute beyond the formal contract requirements. Therefore, in the practice of construction project management, how to cultivate responsible leaders to achieve positive guidance for workers should attract more attention and focus. On the other hand, when researching the benefits brought by responsible leadership, the impact of collective moral sensitivity should be noted. Improving collective moral sensitivity is the key to promote PCB. Project managers can develop collective moral sensitivity of workers through a series of ways such as education and training to improve their sense of moral identity and awareness of social responsibility.

## Limitation and conclusion

### Limitations and future research directions

Several limitations of this study are worth addressing in future research. First, the research design was cross-sectional. The data only described one point in time and cannot reflect dynamic changes. Despite the support of mediation analysis, the direction of causality might be difficult to be fully determined. Future studies should use the method of longitudinal design to properly test the causal relationship, and use multiple data sources to ensure the validation and robustness of the conclusion. Second, although some studies have included other in-role and extra-role rating methods (Arnaud, 2010), most empirical studies still adopt the method of self-rating. In the way of self-rating, employees may tend to overestimate their own performance. Therefore, we believe that future studies could support self-scoring methods by adding additional data, such as peer feedback on an individual’s extra-role contributions (Newman et al., 2017). In addition, the results of this study only reflect the current situation of China. Based on the existing research, the impact of more environmental factors can be explored in the future and further verified in other countries.

A few existing studies have emphasized the role of responsible leadership on OCB, but there have been no studies on exploring how this relationship works from the perspective of project. This study first put forward the influence mechanism of responsible leadership on PCB, and demonstrated what conditions will affect this mechanism from the perspective of project. The results may provide a more in-depth understanding of how leadership affects citizenship behavior in projects for future related research.

## Data availability statement

The raw data supporting the conclusions of this article will be made available by the authors, without undue reservation.

## Ethics statement

Ethical review and approval was not required for the study on human participants in accordance with the local legislation and institutional requirements. Written informed consent from the patients/participants or patients/participants legal guardian/next of kin was not required to participate in this study in accordance with the national legislation and the institutional requirements.

## Author contributions

YY and XZ conceived and designed the research and wrote the manuscript. JH, PW, and SJ conducted the research. JH, HL, and SJ analyzed the data. All authors approved the final version.
